# A systems analysis of biodiesel production from wheat straw using oleaginous yeast: process design, mass and energy balances

**DOI:** 10.1186/s13068-016-0640-9

**Published:** 2016-10-25

**Authors:** Hanna Karlsson, Serina Ahlgren, Mats Sandgren, Volkmar Passoth, Ola Wallberg, Per-Anders Hansson

**Affiliations:** 1 Department of Energy and Technology, Swedish University of Agricultural Sciences, 750 07 Uppsala, Sweden; 2 Department of Chemistry and Biotechnology, Swedish University of Agricultural Sciences, Uppsala, Sweden; 3 Department of Microbiology, Swedish University of Agricultural Sciences, 750 07 Uppsala, Sweden; 4 Department of Chemical Engineering, Lund University, 22100 Lund, Sweden

**Keywords:** Lignocellulosic materials, Diesel, Biogas, Microbial oil, Systems perspective

## Abstract

**Background:**

Biodiesel is the main liquid biofuel in the EU and is currently mainly produced from vegetable oils. Alternative feedstocks are lignocellulosic materials, which provide several benefits compared with many existing feedstocks. This study examined a technical process and its mass and energy balances to gain a systems perspective of combined biodiesel (FAME) and biogas production from straw using oleaginous yeasts. Important process parameters with a determining impact on overall mass and energy balances were identified and evaluated.

**Results:**

In the base case, 41% of energy in the biomass was converted to energy products, primary fossil fuel use was 0.37 MJ_prim_/MJ produced and 5.74 MJ fossil fuels could be replaced per kg straw dry matter. The electricity and heat produced from burning the lignin were sufficient for process demands except in scenarios where the yeast was dried for lipid extraction. Using the residual yeast cell mass for biogas production greatly increased the energy yield, with biogas contributing 38% of total energy products.

**Conclusions:**

In extraction methods without drying the yeast, increasing lipid yield and decreasing the residence time for lipid accumulation are important for the energy and mass balance. Changing the lipid extraction method from wet to dry makes the greatest change to the mass and energy balance. Bioreactor agitation and aeration for lipid accumulation and yeast propagation is energy demanding. Changes in sugar concentration in the hydrolysate and residence times for lipid accumulation greatly affect electricity demand, but have relatively small impacts on fossil energy use (NER) and energy yield (EE). The impact would probably be greater if externally produced electricity were used.

**Electronic supplementary material:**

The online version of this article (doi:10.1186/s13068-016-0640-9) contains supplementary material, which is available to authorized users.

## Background

Use of biodiesel in Sweden has increased more than 11-fold since 2006 [1]. Most (87%) of this biodiesel is either imported or produced from imported feedstock [2]. In EU, biodiesel is the primary liquid biofuel and production increased more than seven-fold from 2003 to 2013 [3]. In Sweden, fatty acid methyl esters (FAME) are produced from rapeseed oil, but other vegetable oils such as soybean oil or palm oil could also be used. Hydrotreated vegetable oils (HVO) are primarily produced from animal fats and tall oil (a by-product from the paper and pulp industry), but also palm oil [2]. Alternative feedstocks for production of biodiesels are lignocellulosic biomass fractions such as straw and forest residues. These feedstocks have several advantages over many of the currently used feedstocks, including good abundance [4], lower cost [5] and lower environmental impact [6]. In addition, agricultural and forest residues do not require extra land and are therefore not associated with competition for food production [7] and indirect land use change. In a Swedish perspective, production of biofuels from lignocellulosic materials presents an opportunity to increase the domestic energy supply security.

Lignocellulosic materials can be converted to fuels and other products through a number of processes that can be roughly categorised into biochemical and thermochemical conversion routes. In biochemical conversion, the biomass is hydrolysed and the resulting sugars can be used as feedstock to produce fuels and chemicals. Biochemical conversion includes lignocellulosic ethanol production and the production of biodiesel using oleaginous yeast, as studied here. In the thermochemical process, the biomass is gasified and the syngas can be catalytically converted to different fuels and chemicals.

Lignocellulosic biomass contains two different types of polysaccharides, cellulose and hemicellulose. When hydrolysed, glucose is obtained from the cellulose, while a mix of pentose and hexose sugars is obtained from the hemicellulose. Ethanol production from lignocellulosic biomass has been extensively studied. Fermentation of pentose sugars to ethanol requires extensive metabolic engineering, because the classical fermentation yeast *Saccharomyces cerevisiae* cannot assimilate these sugars [8]. However, many oleaginous yeasts can use both pentose and hexose sugars for accumulation of lipids [9, 10]. Oleaginous organisms are capable of accumulating more than 20% of their dry weight as lipids [11] and include bacteria, yeasts, filamentous fungi and algae [11, 12]. Yeasts have been identified as promising organisms due to their relatively fast growth rate, ability to grow on multiple substrates (including pentose sugars) at high cell densities, lower risk of viral infection and the option to control bacterial contamination by using low pH conditions [9, 13]. Further upscaling to industrial scale is less complicated for yeasts than for autotrophic microalgae, which have also been considered for biodiesel production [14].

Apart from several technical challenges, industrialisation of biodiesel production from microbial oil (or single cell oil) has been hindered by high fermentation costs, and therefore low-cost lignocellulosic materials have been suggested as feedstock [15]. Low economic profitability of the bulk product, biodiesel, is also hindering the technology from being implemented. Therefore, production of profitable co-products such as animal feed, food or chemicals is vital for the process economics of biodiesel production from lignocellulosic materials [9, 16, 17].

Mass and energy balances of lipid production using oleaginous yeast and different types of yeast and feedstock have been well studied [16, 18–20]. However, apart from a techno-economic analysis studying biodiesel production using glucose as substrate [16], few studies have assessed the energy demand for the entire production processes. So far, no study has assessed mass and energy balances of biodiesel production from lignocellulosic materials in a systems perspective, including feedstock production and transport, biorefinery processing, production of biorefinery inputs and benefits from co-products.

In the present study, a full technical process was studied and its mass and energy balances were calculated to obtain a systems perspective of combined biodiesel (FAME) and biogas production from straw using oleaginous yeast. The aim was to identify and evaluate important process parameters that have a determining impact on overall mass and energy balances.

## Methods

Diesel-like fuels produced from biomass are given various names in the literature, depending on process route, feedstock, etc. In this paper, the term ‘biodiesel’ is used for all diesel-like fuels produced from biomass. When needed, fuel type is specified to FAME, fatty acid ethyl esters (FAEE) and rapeseed methyl esters (RME), hydrated vegetable oils (HVO), dimethyl ether (DME) and Fischer–Tropsch diesel (FT diesel). Lower heating value (LHV) of FAME was assumed to be 37.2 MJ/kg [21].

**Fig. 1:**
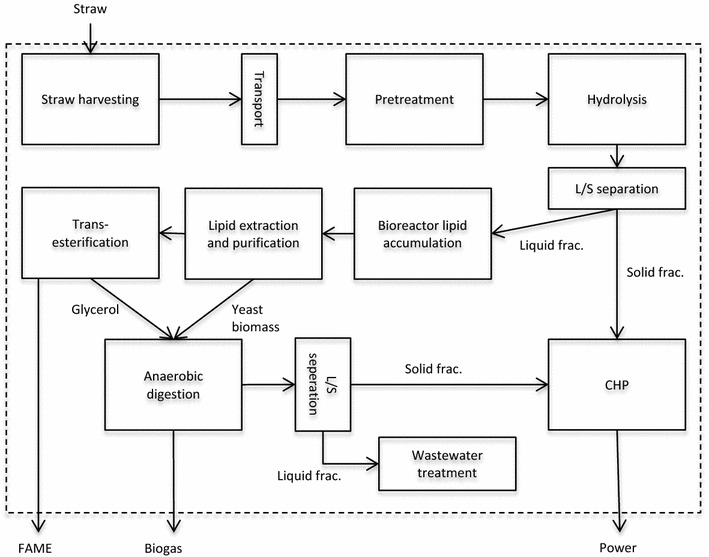
Illustration of the system studied, where the *dotted line* represents the system boundaries

The system studied is illustrated in Fig. 1. Energy and material use during straw harvesting, transport and processing in the biorefinery and biorefinery inputs were included in the analysis.

### Energy balances

A number of indicators of energy systems performance are available [22]. In this study, three different energy performance indicators were used: (1) energy efficiency ratio (EE), calculated as energy produced (LHV)/energy in the feedstock (LHV), indicating the proportion of energy in feedstock converted to final product; (2) net energy ratio (NER), calculated as total primary fossil energy input/energy produced (LHV), indicating the amount of fossil fuel used in production of the biofuel (values >1 indicate more fossil fuel is used than biofuels produced); and (3) an indicator here called fossil fuel replacement potential (FFRP), calculated by subtracting primary fossil fuel potentially replaced by the products from total use of primary energy in the whole production chain for 1 kg of dry matter (DM) straw input into the biorefinery. A positive value of this indicator indicates that more fossil fuel is used in production than biofuels produced, while a negative value indicates the proportion of fossil fuels that could be replaced.

**Fig. 2:**
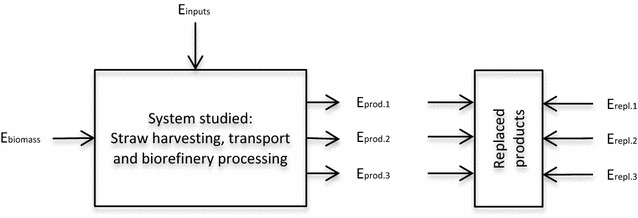
Variables used for the energy balances. *E*_biomass_ and *E*_prod1–3_ are given in LHV and *E*_inputs_ and *E*_repl1–3_ are given in primary fossil energy

The energy balance was calculated as follows (see variables in Fig. 2):

$$EE = (E_{\text{prod1}} + E_{\text{prod2}} + E_{\text{prod3}} )/E_{\text{biomass}}$$

$$NER = E_{\text{inputs}} /(E_{\text{prod1}} + E_{\text{prod2}} + E_{\text{prod3}} )$$

$$FFRP = E_{\text{input}} - (E_{\text{repl1}} + E_{\text{repl2}} + E_{\text{repl3}} )$$

Primary fossil fuel use for the production of inputs and for products replaced by biodiesel, biogas and electricity produced in the biorefinery is presented in Table 1. For all energy balance indicators, biodiesel, biogas and electricity were included as energy products.

**Table 1:** Primary fossil fuel consumption for inputs throughout the production chain and for the products replaced by the products from the biorefinery

	Primary energy use	Reference	Comment
*Input*			
Diesel	1.19 MJ/MJ	[23]	
Acetic acid	53.3 MJ/kg	[24]	98% in water
Enzymes	72.5 MJ/kg product		Personal communication, Jesper Kløverpris, Novozymes A/S, 19 April 2016
Ammonia	41.7 MJ/kg	[24]	
Hexane	60.9 MJ/kg	[24]	
Methanol	37.4 MJ/kg	[24]	
NaOH	42.8 MJ/kg	[24]	50% in water
H_3_PO_4_	23.5 MJ/kg	[24]	85% in water
*Replaced products*			
Biodiesel replaces diesel	1.19 MJ/MJ	[23]	1 MJ biodiesel replaces 1 MJ diesel
Biogas replaces diesel	0.97 MJ/MJ	[23]	1 MJ biogas replaces 0.82 MJ diesel
Electricity replaces natural gas	1.88 MJ/MJ	[23]	

### Straw collection and transport

Collection of straw and loading and unloading from storage was assumed to require 0.27 MJ diesel/kg straw [25–27]. Transport distance for wheat straw in southern Sweden has been estimated at 45 km for a plant processing 120,000 metric tons (t) annually [28] and the same transport distance was assumed for the present study, although the annual straw requirements were substantially lower. Energy use for transport, including empty return, was assumed to be 0.066 MJ/kg straw DM [29].

### Process description—the base case

Process modelling was performed in Aspen Plus™, while Aspen Energy Analyzer™ was used to design the heat exchanger network. The process was modelled as a stand-alone facility with no integration with other processes. The NRTL property method was used for all processes except transesterification, where UNIFAC was used, and combined heat and power (CHP), where STEAM-TA was used. The NREL model for ethanol production from corn stovers [30] described in [31] was used as a basis for modelling the pretreatment, hydrolysis, biogas, wastewater treatment and CHP processes. Detailed information on all unit processes is presented in Additional file 1. Table S1.

The plant was assumed to process 50,000 t wheat straw DM annually. The straw was first comminuted to a particle length of 6 mm, with an estimated energy demand of 9.6 Wh per kg DM [32].

Wheat straw composition was assumed to be glucan (33%), xylan (20%), galactan (1%), arabinan (3%), starch as glucan (3%), acetate (2%), protein (3%), ash (5%), lignin (26%) and extractives (3%) [33]. Pretreatment was performed using steam explosion (190 °C for 10 min) of dilute acid-impregnated wheat straw. Sugar recovery was assumed to be 5% of DM for hexoses and 76% for pentoses, based on recovery rates of glucose and xylose in an earlier publication [33].

Separate hydrolysis and fermentation (SHF) was applied. Use of simultaneous saccharification and fermentation (SSF) for lipid production, which reduces product inhibition during hydrolysis and thereby increases sugar yield, is also described in the literature [17]. However, SHF was selected here due to the major drawbacks of SSF in production of microbial lipids, which have different optimal process conditions for hydrolysis and fermentation, and the presence of unhydrolysed solids in the fermentation, which may make it difficult to extract the lipids [17]. The solid fraction after steam explosion contains the majority of the unhydrolysed cellulose. This fraction was treated with enzymes to hydrolyse the remaining cellulose, with 90% of the cellulose assumed to be hydrolysed [34], while the same hydrolysis rate was assumed for all remaining unhydrolysed hexosans and pentosans. Total sugar recovery from steam explosion and enzymatic hydrolysis was 619 g sugars per kg DM straw (92% of theoretical sugar recovery). The resulting hydrolysate had a sugar content of 150 g/L. Formation of inhibitors was assumed to be 1.7 g HMF and 4.7 g furfural per kg straw [33] giving a final concentration of approx. 0.55 g HMF/L and 1.5 g furfural/L. Studies have shown that oleaginous yeasts tolerate inhibitor concentrations representative of biomass hydrolysates [35]. Many yeast strains grow well with 0.5 g HMF/L, whereas only around 15% of those tested to date grow well with 1 g furfural/L and 25% grow well with 2.5 g acetic acid/L [9, 36]. Acetic acid concentration in the hydrolysate in the present study was approx. 4 g/L. Acetic acid concentrations up to 3.9 g/L have been proven not to be inhibitory for *Lipomyces starkeyi* and, in fact, when present in relatively low concentrations, acetic acid can be used as a carbon source by this yeast [38]. Formation of other acids during steam explosion, such as formic acid, was not accounted for in the present study. It is crucial to find yeast strains that are tolerant to inhibitors, since use of such strains would be more cost-effective than removing the inhibitors [9].

The yeast *Lipomyces starkeyi* was used in this study, since it is reported to be able to assimilate glucose, xylose and acetic acid, which are compounds of lignocellulose hydrolysate. Several studies have shown that this yeast has quite good performance with hydrolysates, with higher lipid concentrations and yields compared with many other yeasts. Moreover, the fatty acids in this yeast showed a high degree of saturation, which is advantageous for biodiesel production [9, 37, 38]. However, it is possible that other yeast species may be more efficient and screening projects to identify optimal strains are ongoing [ [36]; unpublished results]. Lipid accumulation occurs in nitrogen-limited conditions and yeast propagation requires nitrogen. Therefore, yeast propagation and lipid accumulation were performed in separate reactors, with nitrogen added to the yeast reactor in an amount determined stoichiometrically as 5% above the theoretical requirement. The nitrogen in the biomass was assumed to be available to the yeast, with the hydrolysate providing around 11 kg N/h to the yeast propagation reactor, and the additional demand of approximately 80 kg N/h was supplied through addition of ammonia. Residence time was 2 days for yeast propagation and 5 days for lipid accumulation. The fed-batch method was used for lipid accumulation, but downstream processes ran in continuous mode, as the reactors operated in parallel and were refilled and emptied continuously. The volume of yeast propagated was 23.8 m^3^/h and lipid accumulation was 88.4 m^3^/h. Both processes were agitated and aerated with an energy demand of 0.61 kw per m^3^ active volume [39]. The theoretical lipid yield is approximately 0.32 g/g hexose and 0.34 g/g pentose [40]. Actual yields have been estimated to be 0.20–0.22 g lipids/g sugar [40]. In this study, sugar consumption was assumed to be 1.89 g/g yeast cell mass and 3.30 g/g lipids [16]. At the end of the lipid accumulation phase, the lipid content of the cells was assumed to be 50%, giving a final lipid yield of 0.20 g/g sugar, which is slightly lower than the actual yield (0.22 g/g glucose) reported by Jin et al. [17]. Given the residence time and a sugar concentration in the hydrolysate of 150 g/L, the lipid productivity [9] was 0.23 g/L/h.

Lipid extraction and transesterification can be performed either simultaneously or separately. The latter was assumed in this study, as it has been found that the simultaneous process is associated with high costs [16]. In oleaginous yeasts, the lipids are accumulated in lipid bodies, which are lipid bubbles inside the cell [38]. Industrial-scale lipid extraction from yeast is poorly described in the literature. Koutinas et al. [16] proposed a method where the yeast is dried before extraction. However, the drying process is energy-intensive and therefore extraction from wet yeast would be preferable [39]. Extraction from wet yeast is possible at laboratory scale [ [42]; unpublished results]. The oil extraction process was modelled as described elsewhere [41, 43]. The model assumed that the moisture content of the broth was first decreased to 70% using a pressure filter. The cells were then disrupted using a homogeniser, mixed (cascade of five mixers, with a residence time of 600 s per mixer [43]) with hexane used as a solvent (20% w/w yeast in hexane [41]) and the phases separated. Hexane was evaporated and recycled, with hexane losses assumed to be 0.54% [41]. The presence and amount of impurities in the extracted oil are not known for this process. Energy and chemical use for purification were approximated from process descriptions of lipid purification from rapeseed and microalgae oil for biodiesel production [43, 44]. No data were found on losses of lipids during extraction from yeast, but in previous systems analyses of wet lipid extraction from microalgae, losses have been assumed to be 30-2% [41, 43, 45]. In the present study, lipid losses during extraction, solvent recovery and oil purification were assumed to be 10%.

Transesterification was performed using the model of alkali-catalysed biodiesel production from vegetable oil in Aspen Plus [46]. Methanol was used in transesterification of the lipids (average molecular formula C_57_H_104_O_6_) to methyl oleate (C_19_H_36_O_2_). Efficiency of lipid transesterification was assumed to be 98.1% [16]. Quantity of methanol needed was calculated based on a molar ratio of alcohol to lipid of 1:3, corresponding to 0.109 g methanol/g lipid. Glycerol is a by-product from transesterification, but the market for glycerol is saturated, since global production has increased due to increased biodiesel production [43, 47]. Glycerol was therefore fed to the biogas reactor to boost biogas production.

The remaining yeast cell mass after lipid extraction was fed to an anaerobic digestion reactor, methane yield was calculated based on the theoretical yield [48] and efficiency was assumed to be 80% (Åke Nordberg, personal communication 26 Nov. 2015). The biogas was upgraded to 96% (mole fraction) methane content using high-pressure water scrubbing, which was modelled using [49] as a basis. Electricity use was estimated to be 0.32 kWh/m^3^ raw biogas and 0.59 kWh/m^3^ upgraded biogas.

Wastewater treatment with aerobic digestion and a subsequent clarification step was modelled similarly to the process used in NREL [30], with water recirculated into the process. Approximately 32 m^3^ water was needed every hour, of which 24 m^3^ could be recycled. Electricity use for wastewater treatment was approx. 8% of total electricity use at the plant, with the aerobic digestion being the most energy-demanding process (Additional file 1: Table S1).

Lignin and unhydrolysed material from the pretreatment step (as well as small quantities of residues from the anaerobic and aerobic digestions) were combusted in a CHP plant to supply process steam and electricity. The lignin and unhydrolysed material were dewatered using a Pneumapress filter to 50% water content, and the residues from the anaerobic and aerobic digestions were dewatered to 70% water content using a filter press. Energy use for all filters, including associated pumps and compressors, is detailed in Additional file 1: Table S1. The final fuel fed to the combustion had a water content of approx. 51%.

### Scenarios

The following scenarios were analysed:

- Base case as described above.
- Scenario DRY, examining the effect of using the lipid extraction method described in Koutinas et al. [16]: After dewatering in the pressure filter, the yeast was dried to 1% moisture content, then mixed with hexane to 25% (w/w), homogenised, followed by liquid/solid (L/S) separation and finally hexane evaporation and recycling. This scenario was analysed with 10% losses during extraction (DRY10%) and with 5% losses (DRY5%).
- Scenarios LIPID40% and LIPID60%, in which the lipid yield was varied: In the base case lipid concentration in the yeast was assumed to be 50% lipids after 120 h, while in scenarios LIPID40% and LIPID60% it was assumed to be 40 and 60%, respectively, after 120 h.
- Scenarios SUGAR + 10% and SUGAR − 10%, in which the sugar concentration in the hydrolysate was varied by ± 10% from 150 g/L in the base case.
- Scenarios TIME + 1 and TIME − 1, in which the residence time to reach 50% lipid content was varied by ±1 day from the base case.

## Results

For the base case, annual production of biodiesel was 5407 t (55.9 GWh), biogas 2523 t (38.9 GWh) and excess electricity 7.3 GWh. Table 2 shows process inputs and energy demands in the different processing steps. All internal heat demand in the biorefinery was satisfied by combustion of the lignin and other residues from the process. Several processes required cooling, including the reactors for lipid accumulation and yeast propagation, the distillation columns in the transesterification process and the hexane before recycling. The cooling duty for the plant was estimated to be 119 GWh annually. No excess heat was produced, since most processes that required cooling were low-temperature biotechnical processes, mainly the lipid accumulation and yeast propagation reactors, both with a temperature of 25 °C. Agitation and aeration of the reactors during lipid accumulation and yeast propagation was the most energy-demanding process (66% of total electricity use). All power used in the process was supplied from the CHP plant.

**Table 2:** Process inputs per kg DM and per MJ produced (biodiesel, biogas and electricity) and heating demand, given as gross heat demand

	Per kg DM straw	Per MJ produced	Units
*Straw harvesting and transport*			
Diesel (harvesting and transport)	0.34	0.05	MJ
*Pretreatment*			
Electricity	0.07	0.01	MJ
Heat	1.40	0.19	MJ
Sulphuric acid	2.40	0.33	g
*Hydrolysis*			
Electricity	0.06	0.01	MJ
Heat	0.22	0.03	MJ
Enzymes	11.9	1.62	g enzyme product
*Lipid accumulation and yeast propagation*			
Electricity	1.16	0.16	MJ
Heat	0.0	0.00	MJ
Ammonia	13.0	1.77	g
*Lipid extraction and purification*			
Electricity	0.14	0.02	MJ
Heat	0.66	0.09	MJ
Hexane	6.46	0.88	g
Sodium hydroxide	0.32	4.4E−02	g
Phosphoric acid	0.11	1.5E−02	g
*Transesterification*			
Electricity	0.00	0.00	MJ
Heat	0.23	0.03	MJ
Methanol	12.5	1.70	g
Sodium hydroxide	1.02	0.14	g
Phosphoric acid	0.83	0.11	g
*Anaerobic digestion*			
Electricity	0.32	0.04	MJ
Heat	0.13	0.02	MJ

**Fig. 3:**
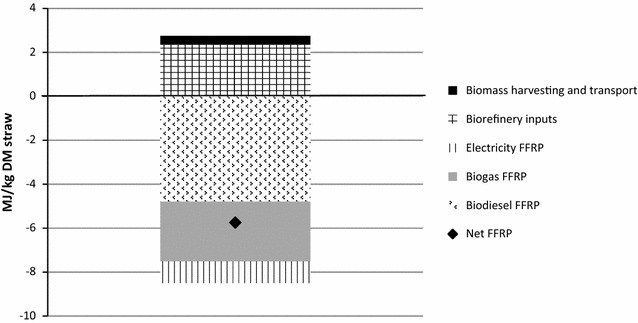
Primary fossil fuel use (positive values) and fossil fuel replacement potential (FFRP) for each product (negative values) for 1 kg straw

Primary fossil fuel use and fossil fuel replacement by the products are presented in Fig. 3 (for 1 kg straw). As in-house energy demand was covered by the CHP plant, fossil energy was only used for production of biorefinery inputs. The largest contributors to fossil fuel use for biorefinery inputs were enzymes for hydrolysing the cellulose (37%), ammonia for yeast propagation (23%), hexane (17%) and methanol (20%). The fossil fuel replacement potential (FFRP) was −5.74 MJ per kg straw (equivalent to −0.32 MJ per MJ LHV straw), meaning that 5.74 MJ primary fossil fuels could be replaced per kg straw processed in the biorefinery (Fig. 2), resulting an annual FFRP of approximately 78.8 GWh. Fossil energy use (NER) was 0.37 MJ_prim_/MJ_product_ and the proportion of the biomass that was converted to an energy carrier (EE) was 41%. Biogas production constituted 38% of total energy production, meaning that biogas production from the residual yeast cell mass and lipid losses during extraction had the potential to substantially increase the energy yield of biodiesel production using oleaginous yeast.

### Scenario analysis

Results for all scenarios, including the base case, are presented in Table 3. Considerable amounts of steam (1.85 MJ/kg straw) were used to dry the yeast (DRY10% and DRY5%), which lowered the electricity production of the CHP plant. Thus instead of generating excess electricity, it needed small external inputs of electricity (0.06 and 0.04 MJ/kg straw for DRY10% and DRY5%, respectively). Furthermore, although biodiesel production increased with decreased losses during lipid extraction (DRY5%), total energy production was very similar between the two scenarios, because the lipids lost in the extraction phase were fed to the biogas reactor and the biogas production in the higher loss (DRY10%) scenario partly compensated for the lipid losses during extraction. The higher NER for the DRY scenarios compared with the base case was due to the lack of excess electricity, the use of external electricity and, in the case of DRY5%, increased use of methanol due to higher biodiesel production. These results show that even if the losses during extraction could be decreased to 5% when the yeast is dried (DRY5%), it would not improve the performance for any of the energy balance indicators compared with the base case with 10% losses. Biogas production from the residues from lipid extraction partly compensated for the decreased biodiesel production and when excess electricity was considered, EE was higher for the base case than for DRY5%.

**Table 3:** Products, major inputs and energy balances for the base case and the eight scenarios studied

	Unit	Base case	DRY10%	DRY5%	LIPID60%	LIPID40%	SUGAR + 10%	SUGAR − 10%	TIME + 1	TIME − 1
*Products*										
Biodiesel	MJ/kg biomass	4.02	4.02	4.25	4.58	3.40	4.02	4.02	4.02	4.02
Biogas	MJ/kg biomass	2.80	2.77	2.56	2.47	3.16	2.80	2.80	2.80	2.80
Excess electricity	MJ/kg biomass	0.52	−0.06	−0.04	0.55	0.49	0.64	0.38	0.32	0.73
Total energy	MJ/kg biomass	7.35	6.79	6.83	7.61	7.06	7.46	7.21	7.15	7.55
*Major process inputs*										
Enzymes	g/kg biomass	11.9	11.9	11.9	11.9	11.9	11.9	11.9	11.9	11.9
Hexane	g/kg biomass	6.46	6.46	6.46	6.14	6.83	6.46	6.46	6.46	6.46
Methanol	g/kg biomass	12.5	12.5	13.2	14.2	10.6	12.5	12.5	12.5	12.5
Ammonia	g/kg biomass	13.0	13.0	13.0	9.9	16.5	13.0	13.0	13.0	13.0
*Energy balances*										
NER	MJ_prim_/MJ_product_	0.37	0.42	0.42	0.35	0.40	0.37	0.38	0.38	0.36
EE	%	41%	38%	38%	43%	39%	42%	40%	40%	42%
FFRP	MJ/kg biomass	−5.74	−4.49	−4.66	−6.22	−5.21	−5.96	−5.48	−5.37	−6.12
*Mass balances*										
Biodiesel	g/kg biomass	108	108	114	123	91	108	108	108	108
Biogas	g/kg biomass	50	50	47	44	57	50	50	50	50

The highest biodiesel production was found for LIPID60%, which assumed 60% lipid content after 120 h residence time in the lipid accumulation reactor. This scenario used less hexane, since total cell mass produced per kg straw, on which hexane use was based, decreased in this scenario. This was because with a lipid content of 60% and yeast cell mass of 40%, more sugars were used for lipid accumulation, which gave lower mass yield than yeast propagation. Furthermore, the lower yeast cell mass content required lower ammonia addition during yeast propagation, while the use of methanol increased due to the higher biodiesel production. Thus overall, the use of fossil energy (NER) decreased by 6% compared with the base case and FFRP increased by 8%.

In the base case, 66% of total electricity was used for agitation and aeration of the reactors for lipid accumulation and yeast propagation. The power demand for agitation and aeration is influenced by lipid productivity (lipids/L/h), i.e. the sugar concentration in the hydrolysate affects the volume of the hydrolysate and the residence times in the reactor, as a lower residence time would mean lower total volume, but also the lipid content in the cells, as described above. Improving the pretreatment in order to increase the sugar concentration of the hydrolysate by 10% (SUGAR + 10%) decreased the NER by 2%, which was due to the higher excess electricity increasing the total amount of energy produced over which total fossil fuel use could be distributed. Similarly, due to the higher excess electricity production, FFRP increased by 4%. Comparable results were obtained on varying the residence time in the lipid accumulation reactor (TIME + 1 and TIME − 1). When the fermentation time was decreased (TIME − 1), excess electricity increased, affecting all energy balance indicators, decreasing NER by 3%, increasing EE by 3% and increasing FFRP by 7%. In both scenarios where the electricity used for agitation and aeration was increased (SUGAR − 10% and TIME + 1), the electricity produced in the CHP plant was still sufficient, although excess electricity decreased. Total electricity use for agitation and aeration increased by 12 and 17% for the SUGAR − 10% and TIME + 1 scenarios, respectively. Impacts on the energy balance indicators would probably be higher if electricity demand exceeded electricity production or if all electricity were sourced externally.

The worst performing scenario for all energy performance indicators was when the yeast was dried with high losses (DRY10%), while the best performing scenario for all energy indicators was when lipid content was increased to 60% after 120 h lipid accumulation (LIPID60%) (Table 3).

## Discussion

Industrial-scale processes for biodiesel production from lignocellulose are poorly described in the literature, apart from single studies (see [16]). This study examined a full technical process and its mass and energy balance for biodiesel production from lignocellulose in a systems perspective.

The highest electricity consumption throughout the process was for aeration and agitation of the bioreactors for yeast propagation and lipid accumulation. Koutinas et al. [16] estimated total electricity use to be 11.3 kWh/kg biodiesel (or 1.09 MJ electricity/MJ biodiesel), with the majority used during agitation and aeration of the bioreactors. In the present study, where the microbubble dispersion technique [39] was applied to save energy, total electricity use was 0.2 MJ/MJ energy product produced (or 0.43 MJ/MJ diesel). The main differences compared with [16] were lower electricity use for agitation and aeration, lower electricity demand for wet lipid extraction instead of dry and production of energy carriers as co-products. The electricity requirement for aeration and agitation is difficult to estimate and is highly influenced by scale. In addition, residence time and sugar concentration in the hydrolysate influence electricity use, and these two factors were varied in the scenario analysis.

In the present study, the sugar concentration in the hydrolysate was assumed to be 150 g/L. It has been argued that the rather low sugar concentration in hydrolysates from lignocellulosic biomass does not favour industrialisation of lipid production using oleaginous yeast [15]. However, some yeast strains have been shown to grow well and accumulate lipids in solutions with sugar concentrations up to 150 g/L [50]. More work is needed to identify yeast strains that can tolerate and accumulate lipids in hydrolysates with high sugar concentrations [9]. Research is ongoing on yeast discovery and genetic engineering for desirable properties such as enhanced or altered lipid production, improved tolerance to inhibitors present in hydrolysate from lignocellulose, growth rate, etc. [9, 17]. The results from the present study showed that increasing the sugar concentration by 10% did not have a large impact on the energy balances. The results would probably have been different if external electricity had been used.

Drying of the yeast requires considerable amounts of energy but has the potential to decrease lipid losses during extraction, which could be beneficial for the overall energy balance. The present study showed that when biogas was produced from process residues, including the lipids lost from lipid extraction, decreasing the losses in the extraction process was not beneficial for any of the energy balance indicators assessed. However, if no alternative use of the lipids is possible, the overall process would most likely benefit from decreasing lipid losses during extraction.

When comparing the energy balance results from this study with findings in previous work, two perspectives are of particular interest. First, biodiesel can be produced from different feedstocks and through different process routes. As diesel is a distinctly different fuel from, e.g. ethanol, it is interesting to compare different biodiesels produced from biomass. Second, it is interesting to compare the mass and energy balances for different process routes to produce transportation fuels from the same or similar feedstock as was used in the present study, namely lignocellulosic materials. Note that results from energy balance studies can sometimes be difficult to compare, as methodologies may differ between studies.

Biodiesel can be produced from different feedstocks, including the vegetable oils that are generally used today, but also other feedstocks such as algae. Studies have shown that production of biodiesel from vegetable oils is associated with fossil fuel use (NER) of 0.15–0.46 MJ_prim_/MJ biodiesel [51, 52]. These fuels, sometimes called first-generation biofuels, are often co-produced with a protein feed (press cake). The energy balance depends strongly on how this co-product is handled in the assessment [51, 52]. In addition, the use of traditional food and feed crops for biofuel production is associated with land use, in contrast to, e.g. straw and forest residues, which are produced without additional land use. Land use and potential indirect land use changes can have large impacts on global warming potential in a life cycle assessment perspective and are therefore important when comparing fuels produced from different feedstocks. Similarly to straw and forest residues, the use of microalgae for lipid accumulation for biodiesel production has been presented as an alternative to first-generation biodiesel. As with biodiesel production from oleaginous yeast, extraction methods for microalgae influence the energy demand of the process and also energy yield, as different extraction methods can be associated with different losses. In one study [53], the fossil energy use (NER) in production of biodiesel using microalgae varied between 0.36 and 3.33 MJ_prim_/MJ biodiesel, with the lower value for wet extraction and the higher for dry extraction.

Different types of fuels can be produced from lignocellulosic biomass, including FT diesel, which is produced thermochemically, or ethanol, which is produced biochemically. Tunå and Hulteberg [54] presented energy balances comparable to the EE indicator used in this study for a number of fuels produced from woody biomass, mainly through the thermochemical process but also ethanol. The estimated EE varied from 66.5% (synthetic natural gas) to 41.2% (ethanol). For the diesel-like fuels, EE was found to be 53% (56.7% including electricity) for DME and 45.6% (51.5% including electricity) for FT diesel [54]. For FT diesel and naphtha and electricity production from corn stovers, EE has been estimated between 43 and 53% [55]. Optimising FT diesel production from switchgrass has been found to yield approx. 12 MJ/kg DM, giving an EE of 68% [56], while combined FT petrol and diesel production from biomass has an EE of 38–39% [57].

The EE values found in this study ranged from 39 to 41% and were thus in the lower range of EE values reported in the studies presented above. However, this indicator does not include energy use during the process, energy use for process inputs and end use of the product, and co-products with no heating value are normally not accounted for. Therefore, additional indicators such as NER and FFRP could add valuable information. Both EE and the NER equate 1 MJ biodiesel to 1 MJ ethanol, which is problematic as driving distance differs for these two fuels. This is accounted for in the FFRP indicator, where 1 MJ ethanol replaces less fossil fuels than 1 MJ diesel when considering driving distance. Although biodiesel has higher FFRP per MJ fuel, a previous study on combined ethanol and biogas production (both pentose and hexose sugars are fermented to ethanol) found a higher FFRP for ethanol (−8.56 MJ) [29] than in the present study (−5.74 MJ in the base case). The higher FFRP was primarily due to higher energy output, but also lower NER value (0.2 MJ_prim_/MJ fuel). The higher fossil fuel use (NER indicator) in the present study was due to the lower energy output (the fossil fuels used were divided over fewer MJ) and higher fossil fuel use during processing, which was mainly due to the ammonia used for yeast cultivation and to methanol and hexane, which are not used in ethanol production. Enzyme production for hydrolysis was the main user of fossil fuels, as is also the case for ethanol [29]. Lignocellulose degrading enzymes are constantly being improved and currently enzyme products with significantly lower energy use for the same conversion efficiency are being introduced (personal communication Jesper Kløverpris, Novozymes A/S, 19 April 2016).

With increasing demand for biodiesel, especially in Europe, it is essential to find alternative feedstocks and production methods to first-generation biodiesel. As described above, lignocellulosic materials have some advantages over many of the currently used feedstocks. Biodiesel from lignocellulose can be produced either through biochemical or thermochemical process routes. It is argued that the thermochemical pathway has lower cost reduction potential, since the FT process has been developed and optimised over a long time, while biochemical ethanol production is less developed [58]. Ethanol production from lignocellulosic biomass is currently gaining considerable attention, while less research is devoted to biodiesel production from lignocellulose. Further research, particularly on process design, including optimal pretreatment and production of valuable by-products such as feed and improvement of yield through strain identification and genetic engineering, could most likely improve the overall energy and mass balance of biodiesel from lignocellulose.

There are currently a number of profit-related challenges in biodiesel production using oleaginous microorganisms, such as low yield, low tolerance to inhibitory compounds from pretreatment, lack of valuable co-products, low concentration and low productivity of lipids, and lack of harvesting and dewatering technologies [9, 17]. Addressing these challenges could also improve the mass and energy balance. This study evaluated biodiesel production as a stand-alone production facility with no integration with other production plants. This led to excessive electricity production, with low energy yield, compared with a case where this excess energy is sold as solid fuel or heat, as the rankine cycle has lower energy efficiency than other alternatives.

Producing biogas from the residual yeast cell mass contributed considerably to energy production. However, cell mass could be used as a nutrient source for the cultivation of yeast, which would decrease the use of nitrogen and thereby improve the energy balance [59]. In addition, the yeast cell mass could be used to generate additional products such as animal or fish feed that could influence the energy and mass balance of the system as a whole. Exploring possible valuable co-products is one way to improve the overall economic profitability and possibly the energy and mass balance. Co-products could include, e.g. essential fatty acids for food applications and oleo-chemicals [17].

In the present study, methanol accounted for 20% of the fossil fuel input. Ethanol could be used instead of methanol in the transesterification process to produce FAME. Co-production of ethanol and biodiesel could be feasible, as hexose sugars can easily be fermented to ethanol, while the pentose sugars could be used for diesel production. Use of ethanol instead of methanol could improve the energy balance and fossil fuel use, as methanol is derived from fossil resources.

Glycerol is a by-product from biodiesel production biodiesel. In this study, the glycerol was fed to the biogas reactor, as the market for glycerol is limited and it was therefore assumed that the glycerol could not be sold. Apart from using the glycerol as feedstock for biogas production, it could also be used as feedstock for biodiesel production using oleaginous yeast [14, 15]. In other words, the glycerol could be fed back to the lipid accumulation reactor to increase biodiesel production and the concentration of substrate in the hydrolysate, with multiple advantages such as decreased energy use during lipid accumulation and lower water content during extraction. The ability of oleaginous yeast to utilise glycerol as a carbon source could also create an alternative market for glycerol from first-generation biodiesel, and co-location of first- and second-generation biodiesel production could give advantages such as direct utilisation of the glycerol produced and combined transesterification.

## Conclusions

This work examined a technical process for biodiesel production from lignocellulose using oleaginous yeast. Energy and mass balance calculations showed that for the base case, fossil energy use (NER) was 0.37 MJ_prim_/MJ and 41% of energy in the initial biomass was converted to an energy carrier in the process (EE). For each kg straw (DM) processed in the plant, approximately 108 g biodiesel and 50.5 g upgraded biogas were produced, together with 0.52 MJ electricity, giving a fossil fuel replacement potential (FFRP) of −5.74 MJ/kg straw. In scenarios involving yeast extraction without drying the yeast, increased lipid yield and decreased residence time for lipid accumulation were important for the energy and mass balance.

Changing the lipid extraction method gave the greatest change in energy and mass balance. Drying the yeast was energy demanding and in scenarios involving this step the energy produced from burning the lignin was not sufficient and the process required some input of external electricity. Although lipid losses were decreased when the yeast was dried, when biogas was produced from process residues, including the lipids lost from lipid extraction, drying the yeast in the extraction process was not beneficial for any of the energy balance indicators calculated.

Agitation and aeration of the bioreactors for lipid accumulation and yeast propagation represented a large proportion of total electricity use in the plant. To decrease the energy demand in the process, more work is needed on energy-efficient agitation and aeration techniques, yeast strains with relatively fast lipid accumulation, and thus decreased residence time, and higher lipid content in cells and pretreatment methods for lignocellulosic materials that yield hydrolysates with high sugar concentrations and low concentrations of inhibitors. Changes in sugar concentration in the hydrolysate and in residence times greatly affected electricity use in the plant, but resulted in relatively small impacts on the NER and EE indicators, although this impact would probably be higher if externally produced electricity were used.

## Additional file

**Additional file 1: Table S1.** Energy demand for unit processes.

## Abbreviations

CHP: combined heat and power
DM: dry matter
DME: dimethyl ether
EE: energy yield
FAEE: fatty acid ethyl esters
FAME: fatty acid methyl esters
FFRP: fossil fuel replacement potential
FT diesel: Fischer–Tropsch diesel
HVO: hydrated vegetable oils
L/S separation: liquid solid separation
LHV: lower heating value
NER: net energy ratio
RME: rapeseed methyl esters
SHF: separate hydrolysis and fermentation
SSF: simultaneous saccharification and fermentation
