# Supplementary material for: A systems analysis of biodiesel production from wheat straw using oleaginous yeast: process design, mass and energy balances
Source: Biotechnol Biofuels. 2016 Oct 25;9:229. doi: 10.1186/s13068-016-0640-9 (PMC5078929; doi:10.1186/s13068-016-0640-9)
Supplement: Supplementary file 1 — Additional file 1: Table S1. Energy demand for unit processes. [file 13068_2016_640_MOESM1_ESM.docx]

Additional file 1. Energy demand for unit processes.

Supporting information for the article: A systems analysis of biodiesel production from wheat straw using oleaginous yeast: Process design, mass and energy balances

Table S1. Energy demand for unit processes with gross heating and cooling requirements (req.)

| **Unit process** | **Electricity req.** | **Heat req.** | **Cooling req.** | **Comment** | **Reference** |
| --- | --- | --- | --- | --- | --- |
| ***Pretreatment*** |  |  |  |  |  |
| Size reduction | 0.0096 kWh/kg biomass (DM)  Total: 60 kW/h |  |  |  | [1] |
| Straw feeding system | 0.0071 kWh/kg straw  Total: 52 kW/h |  |  |  | [2] |
| Pretreatment reactor |  | 0.37 kWh/kg DM straw  Total: 2316 kW/h |  |  | Own calculations |
| Flash | 1.643E-4 kWh/kg inflow  Total: 2.13 kW/h |  |  |  | [2] |
| Heat exchange after pretreatment |  |  | Total: 1668 kW/h |  | Process model |
|  |  |  |  |  |  |
| ***Hydrolysis*** |  |  |  |  |  |
| Pump | Total: 0.58 kW/h |  |  |  | Process model |
| Cooling of hydrolysate |  |  | Total: 337kW/h |  |  |
| Hydrolysis reactor | 0.03 kWh/m3 active volume  Total: 42.7kW/h | Total: 378 kW/h |  |  | Power: [2]  Heat: Process model |
| L/S separation (Pneumpress) | 2.0E-3 kWh/kg solids  Total: 5.38 kW/h |  |  | 2 % losses of soluble sugars to the solid fraction | Power: [2]  Losses: own calculations |
| Pump to L/S separation | Total: 4.47 kW/h |  |  |  | Process model |
| Air compressor for flash dryer | Total: 47.51 kW/h |  |  |  | Process model |
| Cooling air |  |  | Total: 44 kW/h |  | Process model |
|  |  |  |  |  |  |
| ***Lipid accumulation and yeast growth*** |  |  |  |  |  |
| Pump hydrolysate | Total: 1.24 kW/h |  |  |  | Process model |
| Cooling of hydrolysate |  |  | Total: 324 kW/h |  |  |
| Aeration and agitation of fermenters | 0.611 kW/m3 active volume  Total:  Lipid acc: 1746 kW/h  Yeast growth: 260 kW/h |  | Total:  Lipid acc: 3780 kW/h  Yeast growth: 2708 kW/h | Cooling: based on energy content of reactants and energy demand for agitation | Power: [3]  Cooling: own calculations |
| Cooling air |  |  | Total: 90 kW/h |  |  |
| Pump yeast | Total: 0.59 kW/h |  |  |  | Process model |
|  |  |  |  |  |  |
| ***Lipid extraction*** |  |  |  |  |  |
| Pressure filter | 0.88 kWh/m3  Total: 21.2 kW/h |  |  |  | [4] |
| Heating for deactivation of enzymes |  | Total: 195 kW/h |  |  | Process model |
| Homogeniser | 37 kWh/m3  Total: 186 kW/h |  |  |  | [5] |
| Cooling of yeast mass |  |  | Total: 75 kW/h |  | Process model |
| Mixer | 3.3 kW/m3 mixed volume, residence time 50min  Total: 13.8 kW/h |  |  |  | [5] |
| Purification | 30 kWh/ton unpurified oil  Total: 20.2 kW/h | 137 kWh/ton unpurified oil  Total: 92 kW/h |  |  | [6] assuming 90% efficiency of heat boiler |
| Evaporator |  | Total: 860 kW/h |  |  | Process model |
| Cooling recycled hexane |  |  | Total: 895 kW/h |  | Process model |
|  |  |  |  |  |  |
| ***Transesterification*** |  |  |  |  | Process model |
| Pump for lipids | Total: 0.28 kW/h |  |  |  | Process model |
| Pump for methanol | Total: 0.07 kW/h |  |  |  |  |
| Reactor |  |  |  | 99% conversion |  |
| Methanol col. |  | Total: 90 kW/h | Total: 92 kW/h |  | Process model |
| Pump to wash column | Total: 0.09 kW/h |  |  |  | Process model |
| Wash column |  |  |  |  |  |
| Ester column |  | Total: 248 kW/h | Total: 192 kWh/h |  | Process model |
| Glycerol column |  | Total: 61 kW/h | Total: 52 kW/h |  | Process model |
|  |  |  |  |  |  |
| ***WWT including biogas*** |  |  |  | Largely modelled as Area 600 in [2] except for biogas upgrading |  |
| Heater prior to AD |  | Total: 184 kW/h |  |  | Process model |
| Pump2 | Total: 1.98 kW/h |  |  |  | Process model |
| Anaerobic digestion reactor | 0.009 kWh/m3  Total: 35 kW/h | Total: 42 kW/h |  |  | Electricity:[2]  Heat: Process model |
| Pump to filterpress 1 | Total: 6 kW/h |  |  |  | Process model |
| Filterpress 1 | 1.12E-4 kWh/kg inflow  Total: 2.98 kW/h |  |  |  | [2] |
| Aerobic reactor | 0.041 kWh/kg O2 consumed during degradation  Total: 245 kW/h |  | Total: 702 |  | Electricity:[2]  Cooling: Process model |
| Pump | Total: 1.26 kW/h |  |  |  | Process model |
| Clarifier | 2.14E-5 kWh/kg inflow  Total: 0.53 kW/h |  |  |  | Process model |
| Polymer addition | 0,0014 kWh/kg inflow  Total: 1.38 kW/h |  |  |  | [2] |
| Pump to filterpress 2 | 0.44 kW/h |  |  |  | Process model |
| Filterpress 2 | 1.12E-4 kWh/kg inflow  Total: 0.12kW/h |  |  |  | [2] |
| Mechanical cleaning | 4.29 E-5 kWh/kg inflow  Total: 0.011 kW/h |  |  |  | [2] |
| Biogas upgrading | 0.59 kWh/m3 upgraded biogas  Total: 264 kW/h |  | Total: 194 kW/h |  | Own estimations based on [7] |
|  |  |  |  |  |  |
| ***Combustion*** |  |  |  | Modelled as Area 800 in [2] see also [8] | [2, 8] |
| Heat |  | Generated: 4487 kW/h |  |  |  |
| Power | Used: 17.6 kW/h  Generated: 3951 kW/h  Total use in the plant: 3040 kW/h  Excess electricity: 910 kW/h |  |  |  |  |

**References:**

1. Miao Z, Grift TE, Hansen AC, Ting KC. Energy requirement for comminution of biomass in relation to particle physical properties*.* Industrial Crops and Products. 2011;33(2):504-513.

2. NREL. Bioethanol from corn stover process. A sample modell provided by Aspen Tech. National Renewable Energy Laboratory. 2006.

3. Hensirisak P, Parasukulsatid P, Agblevor FA, Cundiff JS, Velander WH. Scale-up of microbubble dispersion generator for aerobic fermentation*.* Applied Biochemistry and Biotechnology. 2002;101(3):211-227.

4. Grima EM,Helarbi E-H, Ferández AFG, Medina RA, Chisti Y. Recovery of microalgal biomass and metabolites: process options and economics. Biotechnology advances. 2003;20(7):491-515.

5. Stephenson AL, Kazamia E, Dennis JS, Howe CJ, Scott SA, Smith AG. Life-cycle assessment of potential algal biodiesel production in the United Kingdom: a comparison of raceways and air-lift tubular bioreactors. Energy & Fuels. 2010;24(7):4062-4077.

6. Stephenson A, Dennis J, Scott S. Improving the sustainability of the production of biodiesel from oilseed rape in the UK. Process Safety and Environmental Protection. 2008;86(6):427-440.

7. Cozma P, Ghinea C, Mămăligă I, Wukovits W, Friedl A, Gavrilescu M. Environmental impact assessment of high pressure water scrubbing biogas upgrading technology. CLEAN–Soil, Air, Water. 2013;41(9):917-927.

8. Humbrid D, Davis R, Tao L, Kinchin C, Hsu D, Aden A, Schoen P, Lukas J, Olthof B, Worley M, Sexton D, Dudgeon D. Process Design and Economics for Biochemical Conversion of Lignocelluloic Biomass to Ethanol Dilute-Acid Pretreatment and enzymatic Hydrolysis of Corn Stover. Technical Report NREL/TP-5100-47764. National Renewable Energy Laboratory. 2011.
